# The Role of Mesenchymal Stem Cells in Regulating Astrocytes-Related Synapse Dysfunction in Early Alzheimer’s Disease

**DOI:** 10.3389/fnins.2022.927256

**Published:** 2022-06-21

**Authors:** Cong Liu

**Affiliations:** NHC and CAMS Key Laboratory of Medical Neurobiology, MOE Frontier Science Center for Brain Research and Brain-Machine Integration, School of Brain Science and Brain Medicine, Zhejiang University, Hangzhou, China

**Keywords:** Alzheimer’s disease, mesenchymal stem cells, astrocytes, amyloid β, neuroinflammation, synaptic functions

## Abstract

Alzheimer’s disease (AD), a neurodegenerative disease, is characterized by the presence of extracellular amyloid-β (Aβ) aggregates and intracellular neurofibrillary tangles formed by hyperphosphorylated tau as pathological features and the cognitive decline as main clinical features. An important cellular correlation of cognitive decline in AD is synapse loss. Soluble Aβ oligomer has been proposed to be a crucial early event leading to synapse dysfunction in AD. Astrocytes are crucial for synaptic formation and function, and defects in astrocytic activation and function have been suggested in the pathogenesis of AD. Astrocytes may contribute to synapse dysfunction at an early stage of AD by participating in Aβ metabolism, brain inflammatory response, and synaptic regulation. While mesenchymal stem cells can inhibit astrogliosis, and promote non-reactive astrocytes. They can also induce direct regeneration of neurons and synapses. This review describes the role of mesenchymal stem cells and underlying mechanisms in regulating astrocytes-related Aβ metabolism, neuroinflammation, and synapse dysfunction in early AD, exploring the open questions in this field.

## Introduction

Alzheimer’s disease (AD) is a neurodegenerative disease, clinically characterized by gradual loss of memory, a decline in cognition, and personality changes. It is the most common cause of dementia among people who are over 65 years old (Long and Holtzman, 2019). According to the age of patients, AD can be divided into two types, that is, early-onset AD (EOAD) and late-onset AD (LOAD). The latter accounts for more than 95% of the total number of AD patients (Mendez, 2017). Multiple factors can lead to AD, but current treatments are not effective enough to cure the patients. Although researchers have studied the disease for over 100 years since it was discovered, there are still controversies concerning the pathology of AD, which is also the main reason why it is hard to cure the disease. With the rapid increase of the aging population, neurodegenerative diseases, like AD, have been listed as highly risky diseases, bringing heavy burdens to families and societies.

Pathological phenotypes of AD include the deposition of amyloid β (Aβ), neurofibrillary tangles (NFTs), neuroinflammation, and synaptic impairment (Mendez, 2017). Aβ plaque has been considered as the major cause of AD pathology, but studies have shown that depletion of the plaque cannot ameliorate dementia (Li and Selkoe, 2020), which indicates that soluble Aβ oligomers may have a closer relationship with AD when compared to those insoluble fibrillary depositions formed by Aβ accumulation. This revised Aβ hypothesis suggests, that the cognitive impairment in AD is mainly caused by the toxicity of soluble Aβ oligomers on synapses. On the cellular level, loss of synapses is an important factor related to cognitive decline (Hardy and Selkoe, 2002) and the cognitive ability of AD patients is positively related to the density of synapses (Palop and Mucke, 2016). Thus, studies have focused on the mechanism concerning the loss of synapses and neurons caused by soluble Aβ oligomers. However, the quantity of synapses does not decline significantly in early AD, and the synaptic dysfunction is preceded by the loss of synapses in the progress of AD (Hardy and Selkoe, 2002), which corresponds with the fact that preclinical AD patients do not have apparent clinical symptoms. Only in the middle or late onset of AD can the loss of synapses be found, but the patients will be hard to cure. Therefore, it will be extremely meaningful to explore the mechanism of synaptic dysfunction in early AD, which will be beneficial to either the diagnosis or the treatment of the disease.

Previously, most studies are focusing on the role of neurons when considering the molecular and cellular mechanism of synaptic dysfunction, but the glia cells cannot be ignored when studying the synaptic impairment induced by Aβ. Astrocyte is the most abundant cells in the central nervous system, participating in several physiological and pathological processes. Astrocyte functions in the formation of synapses, the regulation of synaptic intensity, and the integration of various synaptic activities (Kofuji and Araque, 2021). It can not only monitor the activities of neurons by secreting gliotransmitters such as ATP, glutamate, and D-serine but sense and regulate the synaptic activities as part of “Tripartite” (Kofuji and Araque, 2021). Besides, it can engulf the mature synapses via MEG10 receptor and MERT pathway (Chung et al., 2016). The complement-dependent synapse depletion of microglia is also guided by astrocytes (De Strooper and Karran, 2016). Furthermore, astrocytes are closely related to the metabolism of Aβ (De Strooper and Karran, 2016). Aβ peptide can also affect the phenotypes of astrocytes.

In this regard, it is necessary to find a way to diagnose and cure Alzheimer’s disease in the early stage, and mesenchymal stromal cells (MSCs) might be a target. MSCs are spindle-shaped plastic-adherent cells isolated from bone marrow, adipose, and other tissue sources. They possess an ability of multipotency and immunomodulatory and therefore attract much attention in recent years. Studies have shown that MSCs treatment is not only able to improve neurogenesis and reduce the expression of Aβ related proteins, like β-secretase and γ-secretase, but it can ameliorate either neuroinflammatory conditions by inhibiting the secretion of neuroinflammatory factors from glial cells or synaptic defects by secreting neurotrophic factors. Furthermore, MSCs are also responsible for either the metabolism of glutamate and GABA or the proliferation of neurons and astrocytes. The paper will discuss the role of MSCs in AD treatment mainly from three aspects, that is, Aβ metabolism, astrocyte-induced neuroinflammation, and synaptic defects.

## Mesenchymal Stromal Cells in the Interaction of Aβ Metabolism and Astrocytes

### Astrocytes Functions in Aβ Metabolism

**FIGURE 1:**
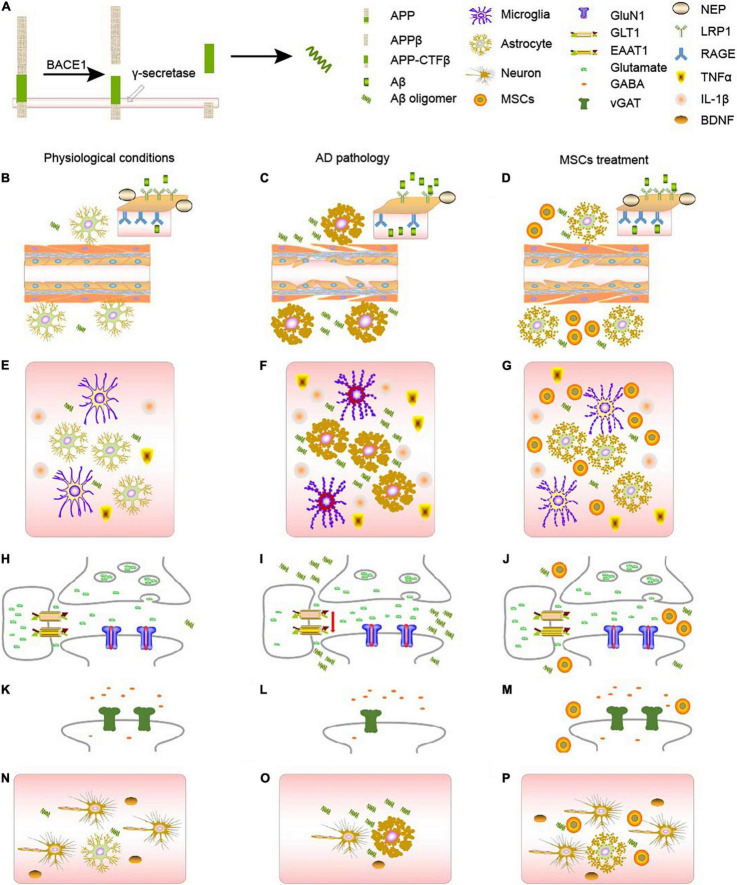
AD pathology and MSCs treatment. **(A)** The generation of Aβ from APP. **(B–D)** Changes of AD metabolism. **(B)** In physiological conditions, APP will gradually be decomposed by BACE1 and γ-secretase. RAGE and LRPs, together with NEP on the blood brain barriers will balance the concentration of Aβ in the body. **(C)** In AD context, astrocytes will become reactive astrocytes, which will disturb the BBB, decreasing the number of RAGE, LRPs, and NEP, and eventually causing the accumulation of Aβ, forming a vicious circle. **(D)** MSCs treatment can turn reactive astrocytes into normal astrocytes, ameliorate the damage of blood vessels, and therefore cure the disease to some extent. **(E–G)** Changes of neuroinflammation. **(E)** In physiological conditions, both astrocytes and microglia will secrete proper amount of inflammatory factors such as TNF-α and IL-1β, maintain the homeostasis in the body. **(F)** In AD context, either astrocytes or microglia will become reactive, which will secrete much more TNF-α and IL-1β. Accumulation of Aβ and reactive microglia will also stimulate astrocytes, aggravating astrogliosis. **(G)** MSCs treatment can turn reactive astrocytes into normal astrocytes, ameliorate the inflammatory context. **(H–J)** Changes of glutamatergic synapses. **(H)** In physiological conditions, astrocytes and neurons form tripartite, regulating the release and clearance of glutamate. **(I)** In AD context, the number of glutamate-related receptors will decrease, especially those on the astrocytes, disturbing the clearance of glutamate. **(J)** MSCs treatment can increase the number of some receptors, and rebalance the glutamate homeostasis. **(K–M)** Changes of GABAergic synapses. **(K)** In physiological conditions, GABA is regulated by vGAT. **(L)** In AD context, the amount of vGAT will decrease, disturbing the GABA homeostasis. **(M)** MSCs treatment can increase the number vGAT, rebalancing the number of GABA in the synapses. **(N–P)** MSCs can ameliorate the damage to neurons through astrocytes. **(N)** In physiological conditions, astrocytes and neurons are together regulating the activities in the brain. And the neurotrophic factors like BDNF, secreted by astrocytes, can support the growth of neurons. **(O)** In AD context, astrocytes will become reactive, there will be less BDNF, and some neurons will die. **(P)** After MSCs treatment, MSCs can turn reactive astrocytes into astrocytes, which can secret neurotrophics like BDNF to support neurons.

Aβ is generated from amyloid β precursor protein via the amyloidogenic processing pathway (Figure 1A). APP will firstly be degraded into soluble APPβ and APP C-terminal fragment β (APP-CTFβ) by β-APP cleaving enzyme (BACE1). APP-CTFβ, also called C99, can be further degraded into Aβ38, Aβ40, or Aβ42 (Sun et al., 2017). Physiological Aβ can maintain homeostasis, synaptic plasticity, and the survival of neurons. Kamenetz et al. (2003) found that the enhancement of BACE1 in the hippocampal brain slices will generate more Aβ peptides, which will inhibit the excitatory synaptic transmission by influencing α-amino-3-hydroxy-5-methyl-4-isoxazolepropionic acid (AMPA) or N-methyl-D-aspartic acid (NMDA).

Studies also suggest that the synaptic activity is positively related to the concentration of Aβ both in APP transgenic mice (Cirrito et al., 2005) and human brains (Brody et al., 2008). Exogenous Aβ can partially block out the metabotropic glutamate receptors (mGluR)-dependent long-term depression (LTD) (Hsieh et al., 2006). Human Aβ1-42 at picomole will enhance the amplitude of long-term potentiation (LTP) in the hippocampus by interacting with the α7-acetylcholine receptor (Puzzo et al., 2008). In the progress of AD, the accumulation of Aβ will form Aβ plaque and induce neurotoxicity, which is mainly caused by the imbalance of the generation and degradation of Aβ (Zulfiqar et al., 2019). Dysfunction of astrocytes will disturb the degradation of Aβ. Reactive astrocytes will release inflammatory factors, induce oxidative stress and eventually lead to the generation and accumulation of Aβ. The interaction between astrocytes and Aβ has widely existed in the brain of AD animal models and patients. In the early AD, the increase of soluble Aβ indicates that the imbalance of Aβ generation and degradation may already exist, and astrocytes may play an important role in the process.

Aβ is mainly cleared up through blood brain barrier (BBB), lymphatic system, and the arachnoid granulation pathway, while the astrocytes can function through the BBB (Cheng et al., 2020). The BBB is comprised of astrocytic endfeet, capillary endothelial cells, and perivascular cells. Aβ transportation through BBB is closely related to two receptors on the endothelial cells, that is, the receptor for advanced glycation end products (RAGE) and lipoprotein receptor-related proteins (LRPs) (Sharma et al., 2012). RAGE is mainly responsible for recruiting exogenous Aβ into the brain, while LRPs can transport the endogenous Aβ to the outside of the brain, together with the insulin-sensitive transporters and ANP-sensitive transporters (Cheng et al., 2020). Thus, it is crucial to maintain the physiological function of astrocytes. Astrocytic senescence, caused by either normal aging or AD pathology, will secret matrix metalloproteinases (MMPs) to induce autophagy of endothelial cells and upregulate the tight junction-related protein to disrupt the junction within endothelial cells, which will impair the BBB and hinder the transportation of Aβ (Han et al., 2020).

Besides, the neprilysin (NEP) within astrocytes can degrade Aβ through the lysosome pathway. Wang et al. (2010) found that the concentration of NEP is negatively related to Aβ accumulation in d patients with no cognitive impairment (NCI) or mild cognitive impairment (MCI), while the relationship disappears among AD patients. Yamamoto et al. (2017) studies the relationship between the secretion of NEP and Aβ degradation in cultured astrocytes and found epigallocatechin gallate (EGCG) will promote the secretion of NEP and induce the degradation of Aβ, probably via extracellular signal-regulated kinase (ERK) or phosphoinositide 3-kinase (PI3K) -mediated pathway.

### Astrocytic Dysfunction Will Aggravate Alzheimer’s Disease Pathology

Now that astrocytes function in the degradation of Aβ induced by protease like NEP, and are mainly relevant in the early AD, astrocytic dysfunction will not only affect the Aβ degradation through BBB (Reeves et al., 2020) but the degradation of Aβ through NEP (Yamamoto et al., 2017). Furthermore, astrocytes can impede the interstitial pulmonary venous drainage, weaken the phagocytosis of microglia, and indirectly lead to the imbalance of Aβ generation and degradation (Kaur et al., 2019; Reeves et al., 2020). Astrocytes can also express apolipoprotein E (ApoE), which can bind to Aβ and make it easier for neurons to intake, and therefore induce the degradation (Arranz and De Strooper, 2019). In the early AD, astrocytic morphology has already changed before the formation of Aβ plaque (Rodríguez-Arellano et al., 2016), which will decrease the secretion of ApoE, and weaken the ability of neurons in terms of Aβ intake (Huynh et al., 2019). This is another reason why the Aβ oligomer will gradually accumulate.

Aβ induces the activation of astrocytes which generate more Aβ. Astrocytes can clear Aβ under physiological conditions, while reactive astrocytes induced by factors like Aβ or LPS can promote the generation of Aβ. Previous studies have shown that BACE1 is only expressed in the neurons, and neurons are therefore considered the unique cells that can generate Aβ (Laird et al., 2005). However, Rossner et al. (2005) point out that BACE1 is not unique to neurons, and they can detect BACE1 on reactive astrocytes. Grolla et al. (2013) not only detected the expression of BACE1 but APP and γ-secretase as well in the rat hippocampal astrocytes. Wang et al. (2019) found toxic Aβ oligomer will replicate in a time-dependent way, leading to the accumulation of Aβ, when overexpressing the BACE1 and ApoE in cultured astrocytes derived from PS1V97L transgenic mice. The phenomenon was not observed in neurons. These results suggest that astrocytes are likely to promote the deposition of Aβ by replicating and spreading the Aβ oligomer and thus expedite the progress of AD.

Besides, astrocytes are involved in the synaptic impairment induced by the Aβ oligomer. Studies have shown that astrocytes can mediate the influence of the Aβ oligomer on synaptic functions in early AD. Diniz et al. (2017) found that the density of synapses will increase when cultured the hippocampal neurons are in an astrocyte-conditional medium (ACM). Apart from the increased density of neurons, the binding of the Aβ oligomer also decreased, and the synaptic loss induced by Aβ was significantly ameliorated. These results indicate that astrocytes can protect the neurons. Astrocytes can secret transforming growth factor-β1 (TGF-β1), which can promote synaptogenesis. If the TGF-β1 was silenced either through neutralizing by antibodies, such as 1D11, or splicing by siRNA, the protective effects of astrocytes will become weaker. TGF-β1 can also ameliorate the loss of hippocampal dendritic spines and memory impairment induced by injecting Aβ into the brain. Astrocytes play a significant role in synaptic impairment induced by Aβ, which is mediated by TGF-β1.

### Mesenchymal Stromal Cells May Ameliorate Astrocyte-Related Defects in Alzheimer’s Disease Pathology

Mesenchymal stromal cells promote functional recoveries in pathological experimental models of the central nervous system (CNS) and are currently being tested in clinical trials for neurological disorders, such as Alzheimer’s disease. Yun et al. (2013) studied the inhibitory effect of placenta-derived MSCs (PD-MSCs) on neuronal cell death and memory impairment in Aβ_1–42_-infused mice. The results showed that the transplantation of PD-MSCs into Aβ_1–42_-infused mice significantly ameliorate cognitive impairment and behavioral changes, and attenuated the expression of APP, BACE1, and enzymes like β-secretase and γ-secretase, all of which are partially regulated by astrocytes. Besides, the paper has discussed the direct role of astrocytes in Aβ metabolism, while MSCs can not only directly target classical AD hallmarks, like Aβ plaque and tau, but they can function through astrocyte-related enzymes, such as neprilysin. Multiple studies using MSCs transplantation in AD transgenic mice models have shown reduced Aβ plaque burden and decreased levels of tau hyperphosphorylation (Naaldijk et al., 2017; Zhao et al., 2018; Santamaria et al., 2021). Soluble intracellular adhesion molecule-1 (sICAM-1) secreted by umbilical cord-derived MSCs can induce the expression of neprilysin (an Aβ-degrading enzyme) and thus facilitate Aβ clearance. MSCs can further reduce the Aβ plaque burden through internalization and Aβ degradation of the endosomal-lysosomal pathway. In animal studies, MSCs transplantation has been shown to ameliorate the symptoms of AD rats by accelerating the clearance of amyloid and tau (Kim et al., 2012). Zhao et al. (2018) also reported Menstrual blood-derived MSCs transplantation dramatically reduced tau phosphorylation at Ser_202_/Thr_205_ (AT_8_) and Ser_396_ sites in the brains of APP/PS1 mice (Zhao et al., 2018).

All in all, astrocytes play an important role in Aβ degradation and astrocytic dysfunction will lead to Aβ deposition. Excess Aβ will turn astrocytes into reactive astrocytes, losing their original functions while further promoting the generation of Aβ, and therefore forming a vicious cycle. Apart from the toxicity of Aβ itself, Aβ can also influence the synaptic function through factors like TGF-β1, which is secreted by astrocytes. And the indirect influence seems to be more crucial when there is no observable loss of neurons, which might be beneficial to the clinical diagnosis of early AD, especially when there are observations about the positive effects from the application of MSCs corresponding to the pathology caused by astrocytic dysfunction.

## Mesenchymal Stromal Cells Ameliorate Astrocyte-Related Neuroinflammation in Alzheimer’s Disease Pathology

### Astrocytes Are Involved in the Neuroinflammation in Alzheimer’s Disease Pathology

Inflammation in the central nervous system has widely existed in many neurodegenerative diseases including AD. Because of the effectiveness of the non-steroidal anti-inflammatory drug (NSAID) in AD treatment, it can be recognized that inflammation is one of the symptoms of AD, and it is likely to be more severe with the progress of AD pathology (Parbo et al., 2017). Parbo et al. (2017) detected the activation of microglia in the brain of MCI patients by using positron emission tomography (PET), which suggests that the inflammatory response has already existed in early AD. Although the activated glial cells can protect neurons to some extent, sometimes it will damage the neurons, either (Parbo et al., 2017). Studies have shown that glial cells are always located around the senile plaques, protecting the neurons by engulfing, degrading, and eliminating Aβ, and therefore limiting the spreading of plaques. However, dysfunction of astrocytes can exacerbate Aβ-related pathology (Lananna et al., 2020). Gulyás et al. (2011) found that L-deprenyl, which is a tracer of astrocytes in PET, has the highest binding rate with astrocytes in Braak I-II patients, and the binding rate will gradually decrease with the progress of diseases. These results suggest that astrocytes play a more important role in early AD. In early AD, soluble Aβ oligomer will gradually accumulate, and its accumulation can not only directly activate astrocytes into reactive astrocytes but promote microglia to secret inflammatory factors as well. These inflammatory factors will further activate astrocytes (Forloni and Balducci, 2018). A1 reactive astrocytes will lose their normal metabolic functions. They will also secret inflammatory factors, making the inflammatory responses severer, which will further activate microglia, and eventually lead to synaptic damage. The expression of the glial fibrillary acid protein (GFAP) in the cerebral cortex and hippocampus of AD patients is significantly increased, which suggests, there are lots of activated astrocytes in the brains.

### A1 Reactive Astrocytes Can Aggravate Inflammatory Context in Alzheimer’s Disease

Reactive astrocytes can be divided into two types, that is, A1 reactive astrocytes and A2 reactive astrocytes. The A1 reactive astrocytes are toxic to the central nervous system, while the A2 reactive astrocytes have a protective effect (Liddelow et al., 2017). Soluble Aβ oligomer can stimulate microglia to secret inflammatory factors like interleukin-1α (IL-1α) and tumor necrosis factor-α (TNF-α), which will activate astrocytes into A1 reactive astrocytes (Liddelow et al., 2017). Although it is still not clear whether Aβ can directly induce astrocytes to become A1 reactive astrocytes, the interaction between Aβ and astrocytes can lead to the proliferation of pro-inflammatory factors and even astrogliosis. The RAGE on the astrocytes is crucial in the process. Soluble RAGE can bind to soluble Aβ, inhibiting the accumulation of Aβ peptides. Membrane-binding RAGE can interact with the Aβ, and activate the nuclear factor κB (NF-κB) pathway, leading to the generation of pro-inflammatory factors such as IL-1β and TNF-α (Gonzalez-Reyes and Rubiano, 2018). In the progress of AD, the imbalance between Aβ generation and degradation will lead to the disruption of RAGE-mediated inhibitive feedback, and therefore increase the concentration of Aβ, activate the NF-κB pathway, and eventually cause chronic inflammation.

Besides, pro-inflammatory factors like IL-1β and TNF-α can promote the astrocytes to secret Aβ. Liao et al. (2004) point out that TNF-α,IL-1β and interferon (IFN-γ) can activate the BACE1 and γ-secretase on the astrocytes through Jun N-terminal kinase (JNK) mitogen-activated protein kinase (JNK-dependent MAPK) signaling pathway, and promote the generation of Aβ. Lee et al. (2008) observed that the concentration of APP and Aβ42 will increase when there is an increase of inflammation-modulating factors such as COX-2, IL-1, and inducible nitric oxide synthase (iNOS) in the LPS-induced inflammation in the central nervous system of mice. These results displayed a close relationship between astrocytic activation and the increase of pro-inflammatory factors.

When treated mice-derived astrocytes with TNF-α and IL-1β, not only the expression of BACE1 and APP will increase, but also the secretion of Aβ_1–40_ (Nixon et al., 1992). Human-derived astrocytes can also secret Aβ_1–40_ and Aβ_1–42_ when exposed to INF-γ and TNF-α or INF-γ and IL-1β (Blasko et al., 2000). All in all, soluble Aβ oligomers can promote the generation of pro-inflammatory factors in early AD and lead to inflammation, while the pro-inflammatory factor can promote the secretion of Aβ even at the physiological concentration, functioning in the balance of Aβ generation and degradation. A vicious cycle will form between the accumulation of Aβ and the secretion of inflammatory factors, both of which can directly affect the neurons, damaging synaptic functions and leading to cognitive impairment.

During the process of the interaction between astrocytes and Aβ, excess proinflammatory factors such as TNF-α, IL-10, and IFN-β can damage synapses. Although there is no direct link revealed between inflammatory factors secreted by A1 reactive astrocytes and the synaptic damage in the current studies concerning AD patients and animal models, the relationship between receptors involving pro-inflammatory factors and the synaptic damage has been widely studied. Studies concerning the dentate gyrus (DG) have shown that TNF-α (600 pmol/L) can activate the type I TNF receptor on the astrocytes, and induce the astrocyte-neuron signaling cascade reaction, leading to the permanent changes in the excitatory synapses in the hippocampus (Habbas et al., 2015). A decrease of IL-10 on astrocytes will weaken their ability to engulf Aβ, and relevant mice models displayed memory impairment in the Morris Water Maze (Zhang et al., 2020). IFN-β can significantly decrease the number of glutamate and aspartate transporter (GLAST) on astrocytes, hindering the transmission of glutamate, while inhibiting the Type I IFN receptor can increase the number of GLAST and positively affect the transfer of glutamate (Hosseini et al., 2020).

### Mesenchymal Stromal Cells Will Ameliorate the Astrocyte-Induced Neuroinflammation

As aforementioned, neuroinflammation plays a pivotal role in the pathogenesis of AD, and astrocytes can aggravate inflammatory conditions. Because mesenchymal stem cells can convert microglia and astrocytes from pro-inflammatory phenotypes M1 and A1 to anti-inflammatory phenotypes M2 and A2, it is believed that MSCs therapy can alleviate the neuroinflammatory response and neuronal damage in AD (Wei et al., 2018; Zhao et al., 2018; Qin et al., 2021). Zhao et al. (2018) recently showed that intracerebral transplantation of menstrual blood-derived MSCs dramatically improved the spatial learning and memory of APP/PS1 mice and the expression of proinflammatory cytokines was remarkably reduced. Xie et al. (2016) reported intravenously transplanted Wharton’s Jelly MSCs significantly improved spatial learning and alleviated the memory decline in the APP/PS1 mice, while the expressions of pro-inflammatory cytokine IL-10 were significantly decreased. In another study, intracerebrally transplanted Adipose-derived MSCs (Ad-MSCs) activated microglia, which decreased expression levels of pro-inflammatory factors and elevated expression levels of Aβ-degrading enzymes, and further reduced β-amyloid (Aβ) peptide deposition as well as significantly restored the learning/memory function in these mice (Ma et al., 2013). Because the decreased number of activated microglia will lead to fewer A1 reactive astrocytes, which will further decrease the expression levels of pro-inflammatory factors and elevate expression levels of anti-inflammatory factors, as well as Aβ-degrading enzymes. These studies suggest MSCs transplantation can alleviate cognitive decline in AD mice through anti-neuroinflammation mechanisms, which are probably regulated by astrocytes.

To sum up, soluble Aβ oligomer can promote the generation of inflammatory factors, causing inflammation in the central nervous system, while pro-inflammatory factors can promote the secretion of Aβ even at physiological concentration, having a crucial role in the balance of Aβ generation and degradation. A vicious cycle will form between the accumulation of Aβ and the secretion of inflammatory factors, during which process microglia, neurons, and astrocytes all play a significant role. Microglia is the initiator of the inflammatory reaction, while astrocytes are the most abundant glial cells. Apart from the toxicity of A1 reactive astrocytes itself, it can also become toxic to neurons, which will aggravate the inflammation. Both the accumulation of Aβ and the increased secretion of pro-inflammatory factors can directly affect the normal function of neurons, weakening their ability to eliminate Aβ, and increasing the release of pro-inflammatory factors, which will lead to cognitive impairment. While it is observed that MSCs may attenuate the inflammatory conditions displayed in AD pathology, mainly by converting the pro-inflammatory phenotype of glia cells into an anti-inflammatory phenotype and eventually ameliorate the pathological conditions of AD patients.

## Astrocytes Can Directly Regulate the Synaptic Functions

### Astrocytes Functions in Synaptic Transmission

Astrocytes widely spread within neurofibril and they enwrap the synapses, forming “tripartite,” together with the presynaptic end and postsynaptic end. They can regulate the synaptic functions by adhering to and sensing neurons, and harmonizing neighboring astrocytes. Astrocytes, located at the perisynapse, can rapidly transfer the neurotransmitters remaining in the synaptic space, by which way they can prevent the accumulation of neurotransmitters outside the synapse and confine them to spread to neighboring synapses (Li and Selkoe, 2020). In addition to communicating with neurons at synaptic levels, astrocytes can integrate into inhibitory neural networks to interact with neurons in neuronal circuits. There are both metabolic and ionic receptors for neurotransmitters on astrocytes, which can regulate the activity of local synaptic circuitry to some extent. Studies have shown that synaptic damages in early AD are most likely caused by Aβ oligomers, rather than insoluble Aβ plaque, and gliotransmitters secreted by astrocytes, such as glutamate, play an important role in the process. With the progress of AD pathology, the soluble Aβ oligomer will increase, disrupting the signaling within the “tripartite,” and eventually causing synaptic dysfunction.

### Astrocyte-Related Synaptic Changes in Alzheimer’s Disease Pathology

#### Aβ and Glutamate

Accompanied by the accumulation of soluble Aβ oligomer and the phosphorylation of tau protein, the level of glutamate outside of cells will gradually increase, causing excitotoxicity and leading to neuronal loss, which is one of the main features of early AD pathology in brains (Zott et al., 2019). Wilcox et al. (2011) point out that synaptic loss, caused by the accumulation of soluble Aβ oligomers, is mediated by the glutamatergic system. When compared to wild-type mice, 2 to 4 – to month-old APP/PS1 mice displays higher excitability in terms of the pyramidal neurons in the hippocampal CA1 region. The number of presynaptic glutamatergic release sites is also upregulated (Hascup and Hascup, 2015). Besides, functional magnetic resonance imaging (fMRI) shows that the whole hippocampus is activated with the deposition of Aβ in patients with mild cognitive impairment (Huijbers et al., 2015). Furthermore, 2 to 6-year-old APP/PS1 mice show an increased release of glutamate (Hascup K. N. et al., 2019), which will be attenuated with the further accumulation of Aβ. Therefore, the astrocytes are significantly related to Aβ pathology by interacting with the glutamate within “tripartite.”

#### Changes of “Tripartite” in Alzheimer’s Disease Pathology

“Tripartite” is comprised of the presynaptic part, postsynaptic part, and perisynaptic part. The presynaptic part and postsynaptic part are considered neuronal components, while the perisynaptic part is comprised of astrocytes, which can help to maintain the homeostasis of synaptic transmission. In AD pathology, astrocytic dysfunction will also affect the neurons, mainly regulated by the changes concerning the release of glutamates, which are induced by the metabolic disturbance of Aβ. As mentioned above, the release of glutamate will increase in early AD, leading to the increased concentration of glutamate within the synapses and its neighboring components. Given the phenomenon that Aβ and vesicular glutamate transporter 1 (VGluT1) are colocalized and are prone to accumulate in the glutamatergic sites, Sokolow et al. (2012) believe that the increase of glutamate concentration starts from presynaptic sites. Furthermore, 2 to 3-month-old APP/PS1 mice have a higher expression of VGluT1 in the hippocampus when compared to wild-type mice, indicating that the transportation of vesicles containing glutamate has increased (Hascup E. R. et al., 2019). The accumulation of Aβ oligomer mainly affects the AMPA receptor and NMDA receptor in the postsynaptic regions. Almeida et al. (2005) cultured the primary neurons derived from the cerebral and hippocampus of Tg2576 transgenic AD mice and found that a higher concentration of Aβ oligomer will lead to lower expression of GluR1, a subunit of AMPA, while the expression of GluA1 is higher when using APP-knockout mice to culture the primary neurons instead of wildtype mice, which suggests that Aβ can directly affect the expression of GluA1 (Martinsson et al., 2019). Studies also showed that the upregulation of AMPA receptors in the hippocampal CA1 region in early AD is caused by the increased release of glutamate in the presynaptic regions. With the progression of AD pathology, the chronic stimulus will lead to the desensitization and internalization of AMPA receptors (Esposito et al., 2013). NMDA is one of the most significant neurotransmitters, relating the accumulation of Aβ to the excitotoxicity of glutamate. Aβ42 is more prone to combine with glutamatergic neurons which express the subunits of NMDA receptors, such as GluN1 and GluN2A or GluN2B (Lacor et al., 2007). Exogenous soluble Aβ with a concentration of 200 nmol/L can promote the release of glutamate, which can further activate the extrasynaptic NMDA receptors through α-7 nicotinic acetylcholine receptors, leading to the inhibition of LTP (Talantova et al., 2013) and the loss of neuron dendritic spine (Kervern et al., 2012). Apart from the neuronal component of the tripartite, the astrocytic component also suffers from significant alterations. Before the formation of senile plaques, the expression of GLAST and glial glutamate transporter 1 (GLT-1) will decrease with the accumulation of Aβ and gliosis, suggesting the attenuated ability of astrocytes concerning glutamate intake (Schallier et al., 2011). This result suggests that the accumulation of glutamate is not only related to the release probability of glutamate in the presynaptic regions but the lower uptake rate of astrocytes as well. Studies have shown that Aβ is likely to affect the expression of glutamate, modifying GLT-1 using lipid peroxidation and 4-hydroxy-nonylphenol of arachidonic acid (4-HEN) (Butterfield et al., 2002). Overexpression of GLT-1 on astrocytes can improve the cognition of 6-month-old APP/PS1 mice, while has little effect on 9-month-old APP/PS1 mice without GLT-1 subunit (Mookherjee et al., 2011). These studies suggest that the dysfunction of astrocytes, which function as perisynaptic components, will disrupt the glutamate metabolism, leading to an increased concentration of glutamate, and affect the synaptic functions. This might be the most important reason why there is a higher concentration of glutamate in the early AD brain. Furthermore, the accumulation of Aβ caused by the attenuated ability of astrocytes in Aβ uptake will further aggravate the above-mentioned effects.

#### Possible Effects of Astrocytic Dysfunction on Other Gliotransmitters

Astrocytes can secret various kinds of gliotransmitters. Apart from glutamate, astrocytes located in the hippocampal CA1 region can also secret D-serine and ATP, both of which are the agonists of NMDA receptors, and are relevant to energy metabolism. Le Douce et al. (2020) found the metabolism of glucose in early AD patients is abnormal in several related brain regions including the hippocampus, which is detected by using fluoro-2-deoxy-D-glucose (FDGa) and PET. Electrophysiological studies suggest that the glycine binding sites of NMDA receptors are significantly decreased in 6 to 7-month-old 3 × Tg-AD mice. And D-serine can rescue the LTP and LTD in the brain slices from the hippocampus of 3 × Tg-AD mice. D-serine is one of the main products of glycolysis, with L-serine as its precursors. Even in early AD, the glycolysis of astrocytes will be abnormal, which indicates that the D-serine is related to the synaptic damage, probably caused by the disrupted pathway for L-serine synthesis (Mookherjee et al., 2011). Besides, ATP is a kind of energy that can be directly used by the human body. In patients with amnestic mild cognitive impairment (aMCI) and AD, oxidative stress will decrease the generation of ATP and attenuate the ability of neurons to maintain the ion concentration gradient, which will inhibit the generation of propagation of action potentials, disrupting the neurotransmission. The imbalance of ion concentration gradient will lead to the influx of cellular Ca2+, triggering the overactivation of Ca2+-dependent nucleic acid enzymes, phospholipase, and protease, eventually causing synaptic dysfunction and even neuronal loss (Butterfield and Halliwell, 2019). Another significant transmitter secreted by astrocytes is γ-aminobutyric acid (GABA), which is the most important inhibitory transmitter in the central nervous system. It is reported that GABA mainly functions in the late AD period. Using an electron microscope, Mitew et al. (2013) observed that the VGlut1 is significantly decreased while vesicular GABA transporter (vGAT) does not change in the hippocampus and cerebral cortex of Braak IV-V AD patients and 12-month-old APP/PS1 mice, while the astrocytes around Aβ plaque will secret more GABA in 12-month-old APP/PS1 mice, which indicate that Aβ plaque may promote astrocytes to secret GABA. Jo et al. (2014) observed an increased secretion of GABA not only in the postmortem tissues of Braak IV-V AD patients but also in the dentate gyrus of 8 to 12-month-old APP/PS1 mice.

To sum up, the alteration of tripartite in the progress of AD is mainly related to glutamate, an excitatory transmitter. Aβ can affect the glutamate-glutamine cycle by regulating the release of glutamate from presences and the intake of glutamate in post-synapses and perisynapses, causing synaptic dysfunction, which is one of the most significant mechanisms of synaptic damage in early AD. Apart from glutamate, the disrupted transmission of other gliotransmitters like D-serine and ATP cannot be ignored. Besides, GABA may also function in the early AD period, although current studies suggest that inhibitory neurotransmitter mainly functions in the late AD period.

### Mesenchymal Stromal Cells Can Ameliorate Synaptic Defects in Alzheimer’s Disease

#### Mesenchymal Stromal Cells and Glutamate Transmission

Glutamate is the main excitatory neurotransmitter in the CNS and increased levels of this neurotransmitter have been associated with a variety of pathological conditions (Kim et al., 2011). The glutamate transporters, EAAT1 and EAAT2, which are mainly expressed in glial cells, have been implicated in the regulation of glutamate levels in the synaptic cleft (Gilley and Kernie, 2011). MSCs can migrate to sites of injury and inflammation and exert therapeutic effects in various neurological disorders, like Alzheimer’s disease. Lee et al. (2014) have examined the ability of MSCs to deliver exogenous miRNA mimics and pre-miRNAs to human neural progenitor cells (NPCs) and astrocytes and characterized the functional impact of this delivery. They found that MSCs efficiently delivered fluorescent-labeled miR-124 and miR-145 mimics to cocultured NPCs and astrocytes. The delivered exogenous miR-124 significantly decreased the expression of the target gene Sox9 by targeting its 3′-UTR and increased the neuronal differentiation of the NPCs. Most importantly, the delivered miR-124 increased the expression of the glutamate transporters EAAT1 and EAAT2 which are expressed in the human NPCs and astrocytes (Lee et al., 2014). So, it is possible to infer that the increased expression of glutamate transporters will ameliorate the excess glutamate in the synaptic cleft and therefore ameliorate the AD pathology.

#### Mesenchymal Stromal Cells and GABAergic Transmission

Studies have found that astrocyte-derived neurotrophins are involved in the regulation of inhibitory transmission and MSCs can release neurotrophins (Elmariah et al., 2005). The stimulation of GABAergic synapses and electrical activity by MSCs could therefore be secondary to the release of proteinaceous neurotrophin(s). This is suggested by the reduced effect of conditioned media from neuron/MSC or neuron/astrocyte co-cultures treated with trypsin. Furthermore, the addition of K252a neurotrophin Trk receptor antagonist (200 nM) to 3 DIV neurons growing in the conditioned media significantly decreased the immunofluorescent staining of vGat-positive versus total synapses in both types of cultures normalized to untreated cultures. This suggests the involvement of neurotrophin(s) acting on the Trk receptor whose identity cannot be established with the K252a experiment. The application of 2 μg/ml TrkB-Fc, which can selectively block BDNF, will also decrease vGAT-positive terminals. Elmariah et al. (2005) also evaluated the BDNF levels in the culture media using an ELISA assay on astrocytes or MSCs individually grown to detect the basal levels of release (pg BDNF normalized to mg proteins). A higher concentration of BDNF in the medium derived from neuron/MSC versus neuron/astrocyte co-cultures was already detected at 3 DIV, with an increment at 10 DIV (pg BDNF normalized to mg proteins). Altogether these results support the role of either astrocytes or MSCs in regulating GABAergic transmission through neurotrophins, which is significant in the process of AD pathology.

#### Mesenchymal Stromal Cells and Neurons Proliferation

Mesenchymal stromal cells are also able to stimulate astrocytic proliferation and then guarantee neuronal survival as de Godoy observed (de Godoy et al., 2018). They found that there is a slightly higher ratio between GFAP-positive and DAPI-positive cells when cocultured astrocytes with MSCs using double immunofluorescence staining of hippocampal cultures, and this phenomenon can maintain for 7 to 10 days. Even when cocultured with neurons, both MSC-conditioned medium and astrocyte-conditioned medium displayed an increment of glial cells. Western Blot and spectrophotometric assays can achieve a similar result (de Godoy et al., 2018). Besides, the increased density of astrocytes proved to be essential for the MSC-promoted survival of hippocampal neurons. Studies have shown that inhibition of astrocyte proliferation in neuronal/MSCs cocultures will lead to the death of neurons, while neurons cultured in an astrocyte-conditioned medium will not suffer, which suggests that the proliferation of glial cells is necessary for the survival of neurons (de Godoy et al., 2018). In the AD context, Aβ oligomers can reduce the level of PSD-95 as analyzed by double immunolabeling (de Godoy et al., 2018). However, when cocultured with MSCs, both PSD-95, a postsynaptic marker, and synaptophysin, a presynaptic marker, tend to increase. Considering the positively interactive relationship between astrocytes and neurons, it is reasonable to infer that MSCs can ameliorate the defects of classical synapses in AD through astrocytes.

Thus, it is possible to tell that MSCs can ameliorate the loss of neurons caused by Aβ in AD pathology by promoting the proliferation of astrocytes, which further emphasizes the importance of astrocytes in the process of AD pathology and the effectiveness of MSCs in the AD therapies.

## Conclusion

Mesenchymal stromal cells have been a hot spot when considering the treatment of several neurological diseases. We discussed the role of MSCs in the process of AD pathology and mainly targeted the astrocyte-related pathologies, such as Aβ metabolism, neuroinflammation, and synaptic functions. Studies have indicated that MSCs can ameliorate the cognitive impairment displayed in AD not only by directly targeted on classical markers of AD, like AD and tau (Figures 1B–D), but also by regulating the neuroinflammatory processes (Figures 1E–G) and synaptic transmission (Figures 1H–P), as summarized in Figure 1. However, there are still some aspects, which need further exploration and elucidation. First, a comprehensive evaluation is required of the source, type, dose, delivery systems, long-term safety and efficacy, the reaction of the implanted cell to the harsh pathogenic AD surroundings, and mechanisms of action in the AD model to confirm their most effective therapeutic results. Second, studies involving MSCs therapies are currently mainly focused on preclinical stages, which cannot guarantee efficacy when applied to the patients. Third, although the pathology of AD is relatively clear than before, lots of efforts are still needed to elucidate different mechanisms. And studies of MSCs may provide a new perspective.

## Author Contributions

CL established the review topic and wrote and edited the manuscript.

## Conflict of Interest

The author declares that the research was conducted in the absence of any commercial or financial relationships that could be construed as a potential conflict of interest.

## Publisher’s Note

All claims expressed in this article are solely those of the authors and do not necessarily represent those of their affiliated organizations, or those of the publisher, the editors and the reviewers. Any product that may be evaluated in this article, or claim that may be made by its manufacturer, is not guaranteed or endorsed by the publisher.
